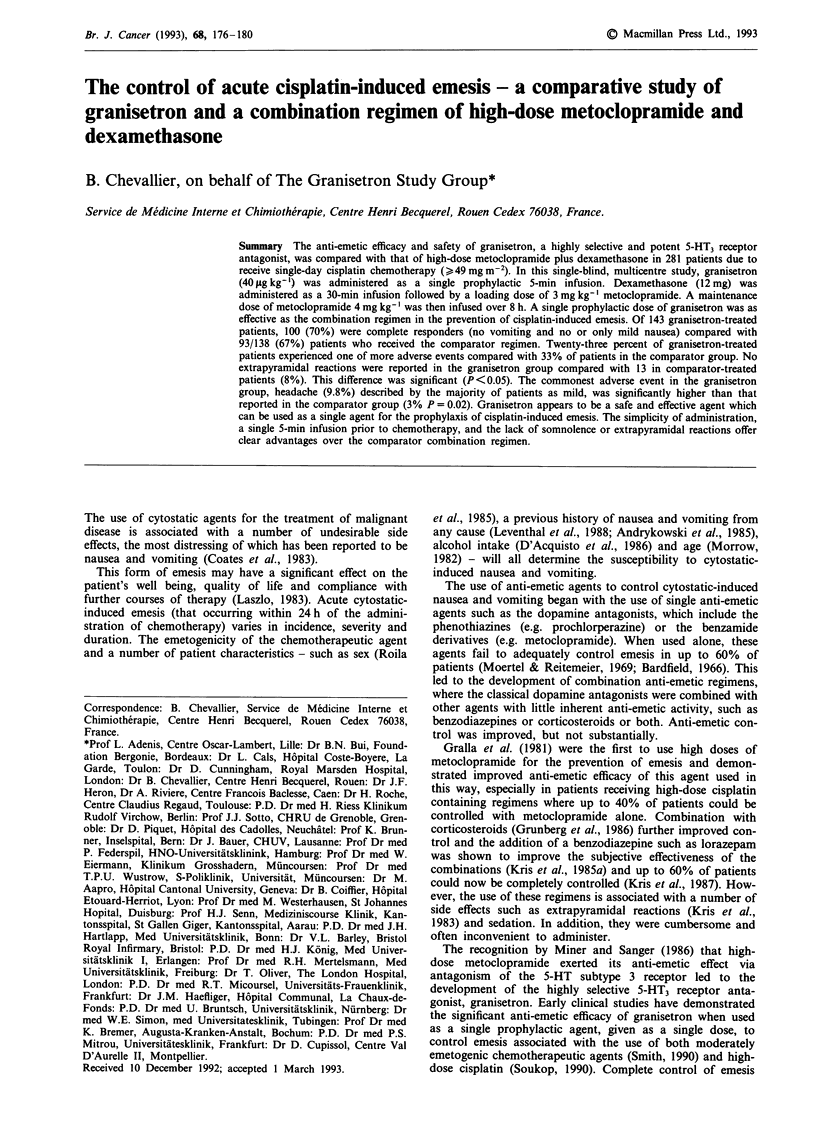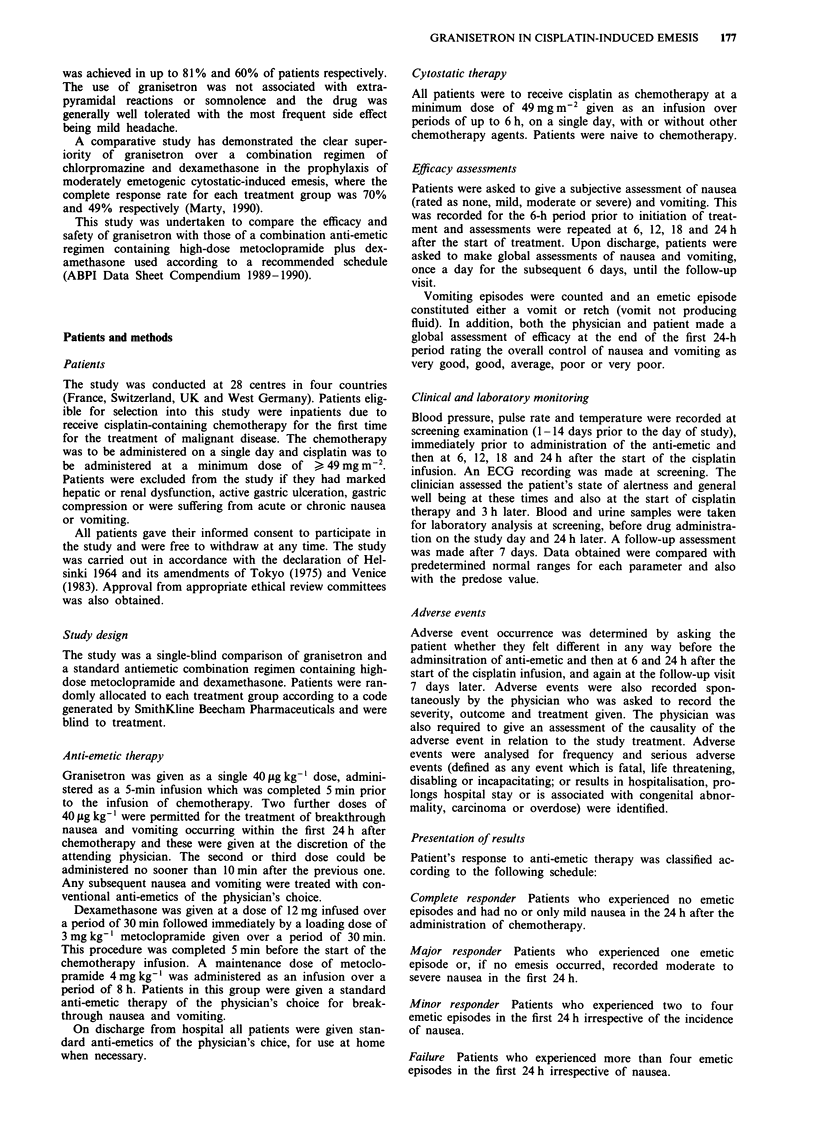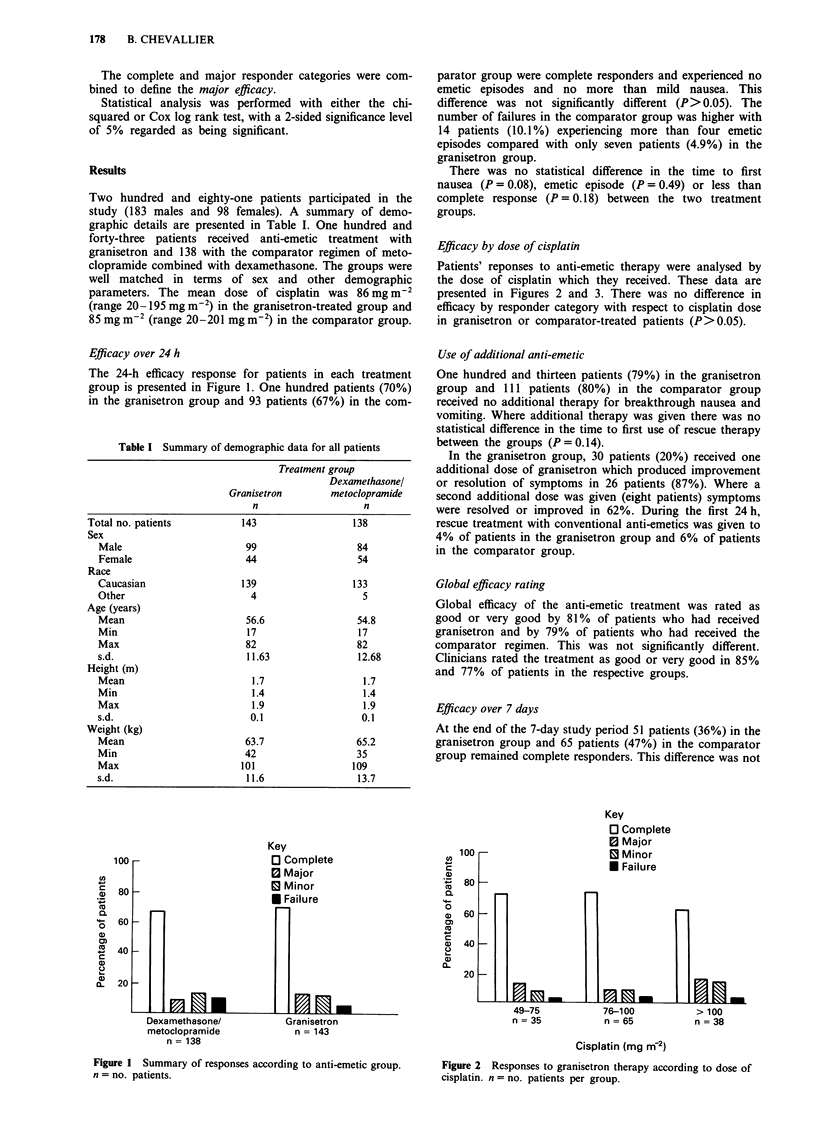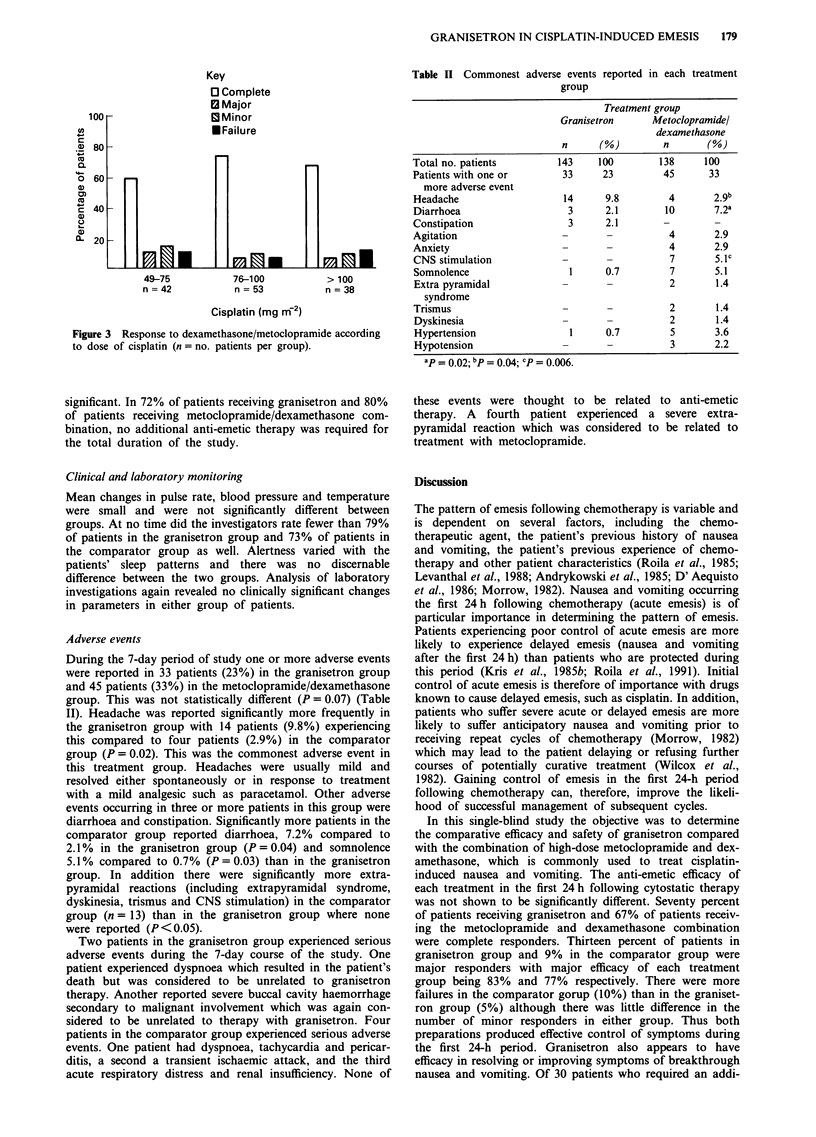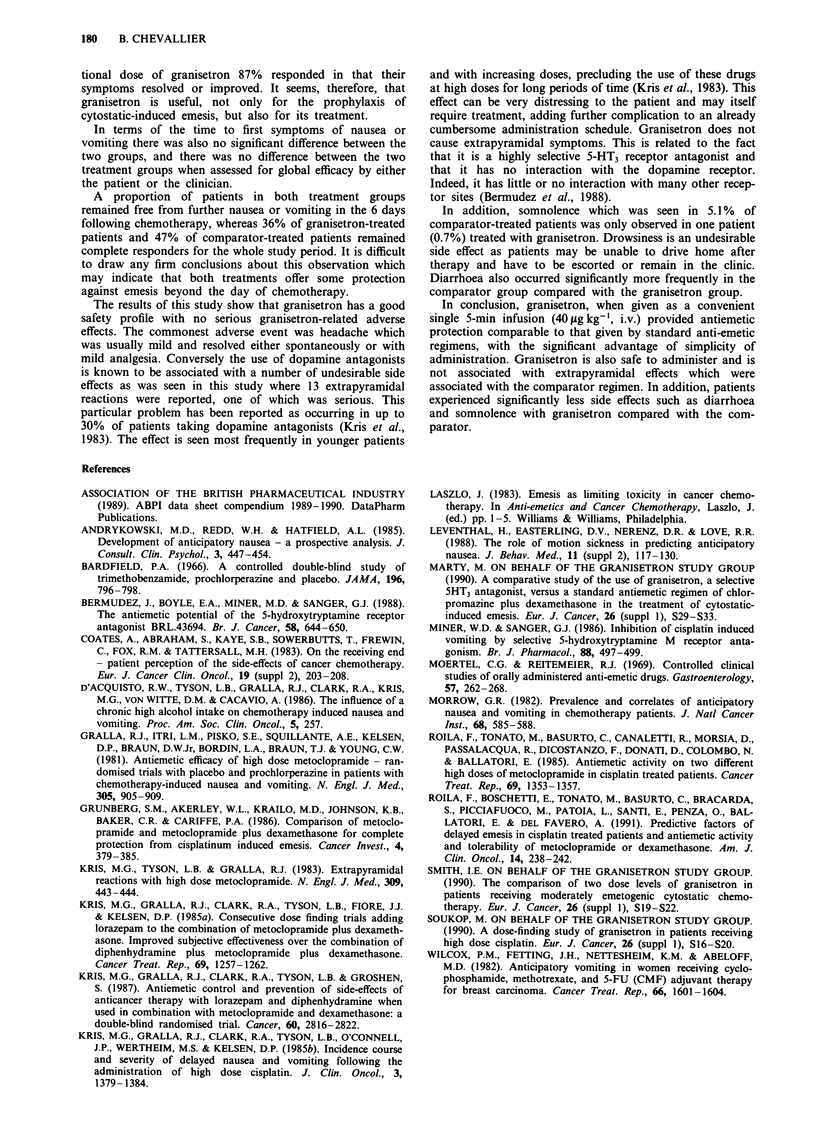# The control of acute cisplatin-induced emesis--a comparative study of granisetron and a combination regimen of high-dose metoclopramide and dexamethasone. Granisetron Study Group.

**DOI:** 10.1038/bjc.1993.309

**Published:** 1993-07

**Authors:** B. Chevallier

**Affiliations:** Service de Médicine Interne et Chimiothérapie, Centre Henri Becquerel, Rouen, France.

## Abstract

The anti-emetic efficacy and safety of granisetron, a highly selective and potent 5-HT3 receptor antagonist, was compared with that of high-dose metoclopramide plus dexamethasone in 281 patients due to receive single-day cisplatin chemotherapy (> or = 49 mg m-2). In this single-blind, multicentre study, granisetron (40 micrograms kg-1) was administered as a single prophylactic 5-min infusion. Dexamethasone (12 mg) was administered as a 30-min infusion followed by a loading dose of 3 mg kg-1 metoclopramide. A maintenance dose of metoclopramide 4 mg kg-1 was then infused over 8 h. A single prophylactic dose of granisetron was as effective as the combination regimen in the prevention of cisplatin-induced emesis. Of 143 granisetron-treated patients, 100 (70%) were complete responders (no vomiting and no or only mild nausea) compared with 93/138 (67%) patients who received the comparator regimen. Twenty-three percent of granisetron-treated patients experienced one of more adverse events compared with 33% of patients in the comparator group. No extrapyramidal reactions were reported in the granisetron group compared with 13 in comparator-treated patients (8%). This difference was significant (P < 0.05). The commonest adverse event in the granisetron group, headache (9.8%) described by the majority of patients as mild, was significantly higher than that reported in the comparator group (3% P = 0.02). Granisetron appears to be a safe and effective agent which can be used as a single agent for the prophylaxis of cisplatin-induced emesis. The simplicity of administration, a single 5-min infusion prior to chemotherapy, and the lack of somnolence or extrapyramidal reactions offer clear advantages over the comparator combination regimen.


*Br. J. Cancer* (1993), **68**, 176–180 © Macmillan Press Ltd., 1993

### The control of acute cisplatin-induced emesis – a comparative study of granisetron and a combination regimen of high-dose metoclopramide and dexamethasone

B. Chevallier, on behalf of The Granisetron Study Group*

*Service de Médicine Interne et Chimiothérapie, Centre Henri Becquerel, Rouen Cedex 76038, France.*

**Summary** The anti-emetic efficacy and safety of granisetron, a highly selective and potent 5-HT$_3$ receptor antagonist, was compared with that of high-dose metoclopramide plus dexamethasone in 281 patients due to receive single-day cisplatin chemotherapy ($\geqslant$49 mg m$^{-2}$). In this single-blind, multicentre study, granisetron (40 $\mu$g kg$^{-1}$) was administered as a single prophylactic 5-min infusion. Dexamethasone (12 mg) was administered as a 30-min infusion followed by a loading dose of 3 mg kg$^{-1}$ metoclopramide. A maintenance dose of metoclopramide 4 mg kg$^{-1}$ was then infused over 8 h. A single prophylactic dose of granisetron was as effective as the combination regimen in the prevention of cisplatin-induced emesis. Of 143 granisetron-treated patients, 100 (70%) were complete responders (no vomiting and no or only mild nausea) compared with 93/138 (67%) patients who received the comparator regimen. Twenty-three percent of granisetron-treated patients experienced one of more adverse events compared with 33% of patients in the comparator group. No extrapyramidal reactions were reported in the granisetron group compared with 13 in comparator-treated patients (8%). This difference was significant ($P<0.05$). The commonest adverse event in the granisetron group, headache (9.8%) described by the majority of patients as mild, was significantly higher than that reported in the comparator group (3% $P = 0.02$). Granisetron appears to be a safe and effective agent which can be used as a single agent for the prophylaxis of cisplatin-induced emesis. The simplicity of administration, a single 5-min infusion prior to chemotherapy, and the lack of somnolence or extrapyramidal reactions offer clear advantages over the comparator combination regimen.

The use of cytostatic agents for the treatment of malignant disease is associated with a number of undesirable side effects, the most distressing of which has been reported to be nausea and vomiting (Coates *et al.*, 1983).

This form of emesis may have a significant effect on the patient's well being, quality of life and compliance with further courses of therapy (Laszlo, 1983). Acute cytostatic-induced emesis (that occurring within 24 h of the administration of chemotherapy) varies in incidence, severity and duration. The emetogenicity of the chemotherapeutic agent and a number of patient characteristics – such as sex (Roila *et al.*, 1985), a previous history of nausea and vomiting from any cause (Leventhal *et al.*, 1988; Andrykowski *et al.*, 1985), alcohol intake (D'Acquisto *et al.*, 1986) and age (Morrow, 1982) – will all determine the susceptibility to cytostatic-induced nausea and vomiting.

The use of anti-emetic agents to control cytostatic-induced nausea and vomiting began with the use of single anti-emetic agents such as the dopamine antagonists, which include the phenothiazines (e.g. prochlorperazine) or the benzamide derivatives (e.g. metoclopramide). When used alone, these agents fail to adequately control emesis in up to 60% of patients (Moertel & Reitemeier, 1969; Bardfield, 1966). This led to the development of combination anti-emetic regimens, where the classical dopamine antagonists were combined with other agents with little inherent anti-emetic activity, such as benzodiazepines or corticosteroids or both. Anti-emetic control was improved, but not substantially.

Gralla *et al.* (1981) were the first to use high doses of metoclopramide for the prevention of emesis and demonstrated improved anti-emetic efficacy of this agent used in this way, especially in patients receiving high-dose cisplatin containing regimens where up to 40% of patients could be controlled with metoclopramide alone. Combination with corticosteroids (Grunberg *et al.*, 1986) further improved control and the addition of a benzodiazepine such as lorazepam was shown to improve the subjective effectiveness of the combinations (Kris *et al.*, 1985*a*) and up to 60% of patients could now be completely controlled (Kris *et al.*, 1987). However, the use of these regimens is associated with a number of side effects such as extrapyramidal reactions (Kris *et al.*, 1983) and sedation. In addition, they were cumbersome and often inconvenient to administer.

The recognition by Miner and Sanger (1986) that high-dose metoclopramide exerted its anti-emetic effect via antagonism of the 5-HT subtype 3 receptor led to the development of the highly selective 5-HT$_3$ receptor antagonist, granisetron. Early clinical studies have demonstrated the significant anti-emetic efficacy of granisetron when used as a single prophylactic agent, given as a single dose, to control emesis associated with the use of both moderately emetogenic chemotherapeutic agents (Smith, 1990) and high-dose cisplatin (Soukop, 1990). Complete control of emesis

Correspondence: B. Chevallier, Service de Médicine Interne et Chimiothérapie, Centre Henri Becquerel, Rouen Cedex 76038, France.

*Prof L. Adenis, Centre Oscar-Lambert, Lille: Dr B.N. Bui, Foundation Bergonie, Bordeaux: Dr L. Cals, Hôpital Coste-Boyere, La Garde, Toulon: Dr D. Cunningham, Royal Marsden Hospital, London: Dr B. Chevallier, Centre Henri Becquerel, Rouen: Dr J.F. Heron, Dr A. Riviere, Centre Francois Baclesse, Caen: Dr H. Roche, Centre Claudius Regaud, Toulouse: P.D. Dr med H. Riess Klinikum Rudolf Virchow, Berlin: Prof J.J. Sotto, CHRU de Grenoble, Grenoble: Dr D. Piquet, Hôpital des Cadolles, Neuchâtel: Prof K. Brunner, Inselspital, Bern: Dr J. Bauer, CHUV, Lausanne: Prof Dr med P. Federspil, HNO-Universitätsklinink, Hamburg: Prof Dr med W. Eiermann, Klinikum Grosshadern, Müncoursen: Prof Dr med T.P.U. Wustrow, S-Poliklinik, Universität, Müncoursen: Dr M. Aapro, Hôpital Cantonal University, Geneva: Dr B. Coiffier, Hôpital Etouard-Herriot, Lyon: Prof Dr med M. Westerhausen, St Johannes Hopital, Duisburg: Prof H.J. Senn, Medizinicourse Klinik, Kantonsspital, St Gallen Giger, Kantonsspital, Aarau: P.D. Dr med J.H. Hartlapp, Med Universitätsklinik, Bonn: Dr V.L. Barley, Bristol Royal Infirmary, Bristol: P.D. Dr med H.J. König, Med Universitätsklinik I, Erlangen: Prof Dr med R.H. Mertelsmann, Med Universitätsklinik, Freiburg: Dr T. Oliver, The London Hospital, London: P.D. Dr med R.T. Micoursel, Universitäts-Frauenklinik, Frankfurt: Dr J.M. Haefliger, Hôpital Communal, La Chaux-de-Fonds: P.D. Dr med U. Bruntsch, Universitätsklinik, Nürnberg: Dr med W.E. Simon, med Universitatesklinik, Tubingen: Prof Dr med K. Bremer, Augusta-Kranken-Anstalt, Bochum: P.D. Dr med P.S. Mitrou, Universitätesklinik, Frankfurt: Dr D. Cupissol, Centre Val D'Aurelle II, Montpellier.

Received 10 December 1992; accepted 1 March 1993.

was achieved in up to 81% and 60% of patients respectively. The use of granisetron was not associated with extra-pyramidal reactions or somnolence and the drug was generally well tolerated with the most frequent side effect being mild headache.

A comparative study has demonstrated the clear superiority of granisetron over a combination regimen of chlorpromazine and dexamethasone in the prophylaxis of moderately emetogenic cytostatic-induced emesis, where the complete response rate for each treatment group was 70% and 49% respectively (Marty, 1990).

This study was undertaken to compare the efficacy and safety of granisetron with those of a combination anti-emetic regimen containing high-dose metoclopramide plus dexamethasone used according to a recommended schedule (ABPI Data Sheet Compendium 1989–1990).

### Patients and methods

### *Patients*

The study was conducted at 28 centres in four countries (France, Switzerland, UK and West Germany). Patients eligible for selection into this study were inpatients due to receive cisplatin-containing chemotherapy for the first time for the treatment of malignant disease. The chemotherapy was to be administered on a single day and cisplatin was to be administered at a minimum dose of $\geqslant 49\,\mathrm{mg\,m^{-2}}$. Patients were excluded from the study if they had marked hepatic or renal dysfunction, active gastric ulceration, gastric compression or were suffering from acute or chronic nausea or vomiting.

All patients gave their informed consent to participate in the study and were free to withdraw at any time. The study was carried out in accordance with the declaration of Helsinki 1964 and its amendments of Tokyo (1975) and Venice (1983). Approval from appropriate ethical review committees was also obtained.

### *Study design*

The study was a single-blind comparison of granisetron and a standard antiemetic combination regimen containing high-dose metoclopramide and dexamethasone. Patients were randomly allocated to each treatment group according to a code generated by SmithKline Beecham Pharmaceuticals and were blind to treatment.

### *Anti-emetic therapy*

Granisetron was given as a single $40\,\mu\mathrm{g\,kg^{-1}}$ dose, administered as a 5-min infusion which was completed 5 min prior to the infusion of chemotherapy. Two further doses of $40\,\mu\mathrm{g\,kg^{-1}}$ were permitted for the treatment of breakthrough nausea and vomiting occurring within the first 24 h after chemotherapy and these were given at the discretion of the attending physician. The second or third dose could be administered no sooner than 10 min after the previous one. Any subsequent nausea and vomiting were treated with conventional anti-emetics of the physician's choice.

Dexamethasone was given at a dose of 12 mg infused over a period of 30 min followed immediately by a loading dose of $3\,\mathrm{mg\,kg^{-1}}$ metoclopramide given over a period of 30 min. This procedure was completed 5 min before the start of the chemotherapy infusion. A maintenance dose of metoclopramide $4\,\mathrm{mg\,kg^{-1}}$ was administered as an infusion over a period of 8 h. Patients in this group were given a standard anti-emetic therapy of the physician's choice for breakthrough nausea and vomiting.

On discharge from hospital all patients were given standard anti-emetics of the physician's chice, for use at home when necessary.

### *Cytostatic therapy*

All patients were to receive cisplatin as chemotherapy at a minimum dose of $49\,\mathrm{mg\,m^{-2}}$ given as an infusion over periods of up to 6 h, on a single day, with or without other chemotherapy agents. Patients were naive to chemotherapy.

### *Efficacy assessments*

Patients were asked to give a subjective assessment of nausea (rated as none, mild, moderate or severe) and vomiting. This was recorded for the 6-h period prior to initiation of treatment and assessments were repeated at 6, 12, 18 and 24 h after the start of treatment. Upon discharge, patients were asked to make global assessments of nausea and vomiting, once a day for the subsequent 6 days, until the follow-up visit.

Vomiting episodes were counted and an emetic episode constituted either a vomit or retch (vomit not producing fluid). In addition, both the physician and patient made a global assessment of efficacy at the end of the first 24-h period rating the overall control of nausea and vomiting as very good, good, average, poor or very poor.

### *Clinical and laboratory monitoring*

Blood pressure, pulse rate and temperature were recorded at screening examination (1–14 days prior to the day of study), immediately prior to administration of the anti-emetic and then at 6, 12, 18 and 24 h after the start of the cisplatin infusion. An ECG recording was made at screening. The clinician assessed the patient's state of alertness and general well being at these times and also at the start of cisplatin therapy and 3 h later. Blood and urine samples were taken for laboratory analysis at screening, before drug administration on the study day and 24 h later. A follow-up assessment was made after 7 days. Data obtained were compared with predetermined normal ranges for each parameter and also with the predose value.

### *Adverse events*

Adverse event occurrence was determined by asking the patient whether they felt different in any way before the adminsitration of anti-emetic and then at 6 and 24 h after the start of the cisplatin infusion, and again at the follow-up visit 7 days later. Adverse events were also recorded spontaneously by the physician who was asked to record the severity, outcome and treatment given. The physician was also required to give an assessment of the causality of the adverse event in relation to the study treatment. Adverse events were analysed for frequency and serious adverse events (defined as any event which is fatal, life threatening, disabling or incapacitating; or results in hospitalisation, prolongs hospital stay or is associated with congenital abnormality, carcinoma or overdose) were identified.

### *Presentation of results*

Patient's response to anti-emetic therapy was classified according to the following schedule:

*Complete responder* Patients who experienced no emetic episodes and had no or only mild nausea in the 24 h after the administration of chemotherapy.

*Major responder* Patients who experienced one emetic episode or, if no emesis occurred, recorded moderate to severe nausea in the first 24 h.

*Minor responder* Patients who experienced two to four emetic episodes in the first 24 h irrespective of the incidence of nausea.

*Failure* Patients who experienced more than four emetic episodes in the first 24 h irrespective of nausea.

The complete and major responder categories were combined to define the *major efficacy*.

Statistical analysis was performed with either the chi-squared or Cox log rank test, with a 2-sided significance level of 5% regarded as being significant.

### Results

Two hundred and eighty-one patients participated in the study (183 males and 98 females). A summary of demographic details are presented in Table I. One hundred and forty-three patients received anti-emetic treatment with granisetron and 138 with the comparator regimen of metoclopramide combined with dexamethasone. The groups were well matched in terms of sex and other demographic parameters. The mean dose of cisplatin was 86 mg m$^{-2}$ (range 20–195 mg m$^{-2}$) in the granisetron-treated group and 85 mg m$^{-2}$ (range 20–201 mg m$^{-2}$) in the comparator group.

### *Efficacy over 24 h*

The 24-h efficacy response for patients in each treatment group is presented in Figure 1. One hundred patients (70%) in the granisetron group and 93 patients (67%) in the comparator group were complete responders and experienced no emetic episodes and no more than mild nausea. This difference was not significantly different ($P>0.05$). The number of failures in the comparator group was higher with 14 patients (10.1%) experiencing more than four emetic episodes compared with only seven patients (4.9%) in the granisetron group.

There was no statistical difference in the time to first nausea ($P=0.08$), emetic episode ($P=0.49$) or less than complete response ($P=0.18$) between the two treatment groups.

**Table I** Summary of demographic data for all patients

| | Treatment group | |
|---|---|---|
| | Granisetron *n* | Dexamethasone/ metoclopramide *n* |
| Total no. patients | 143 | 138 |
| Sex | | |
| Male | 99 | 84 |
| Female | 44 | 54 |
| Race | | |
| Caucasian | 139 | 133 |
| Other | 4 | 5 |
| Age (years) | | |
| Mean | 56.6 | 54.8 |
| Min | 17 | 17 |
| Max | 82 | 82 |
| s.d. | 11.63 | 12.68 |
| Height (m) | | |
| Mean | 1.7 | 1.7 |
| Min | 1.4 | 1.4 |
| Max | 1.9 | 1.9 |
| s.d. | 0.1 | 0.1 |
| Weight (kg) | | |
| Mean | 63.7 | 65.2 |
| Min | 42 | 35 |
| Max | 101 | 109 |
| s.d. | 11.6 | 13.7 |

### *Efficacy by dose of cisplatin*

Patients' reponses to anti-emetic therapy were analysed by the dose of cisplatin which they received. These data are presented in Figures 2 and 3. There was no difference in efficacy by responder category with respect to cisplatin dose in granisetron or comparator-treated patients ($P>0.05$).

### *Use of additional anti-emetic*

One hundred and thirteen patients (79%) in the granisetron group and 111 patients (80%) in the comparator group received no additional therapy for breakthrough nausea and vomiting. Where additional therapy was given there was no statistical difference in the time to first use of rescue therapy between the groups ($P=0.14$).

In the granisetron group, 30 patients (20%) received one additional dose of granisetron which produced improvement or resolution of symptoms in 26 patients (87%). Where a second additional dose was given (eight patients) symptoms were resolved or improved in 62%. During the first 24 h, rescue treatment with conventional anti-emetics was given to 4% of patients in the granisetron group and 6% of patients in the comparator group.

### *Global efficacy rating*

Global efficacy of the anti-emetic treatment was rated as good or very good by 81% of patients who had received granisetron and by 79% of patients who had received the comparator regimen. This was not significantly different. Clinicians rated the treatment as good or very good in 85% and 77% of patients in the respective groups.

### *Efficacy over 7 days*

At the end of the 7-day study period 51 patients (36%) in the granisetron group and 65 patients (47%) in the comparator group remained complete responders. This difference was not

**Figure 1** Summary of responses according to anti-emetic group. *n* = no. patients.

**Figure 2** Responses to granisetron therapy according to dose of cisplatin. *n* = no. patients per group.

**Figure 3** Response to dexamethasone/metoclopramide according to dose of cisplatin ($n$ = no. patients per group).

**Table II** Commonest adverse events reported in each treatment group

| | *Treatment group* | | | |
|---|---|---|---|---|
| | *Granisetron* | | *Metoclopramide/ dexamethasone* | |
| | *n* | *(%)* | *n* | *(%)* |
| Total no. patients | 143 | 100 | 138 | 100 |
| Patients with one or more adverse event | 33 | 23 | 45 | 33 |
| Headache | 14 | 9.8 | 4 | 2.9[b] |
| Diarrhoea | 3 | 2.1 | 10 | 7.2[a] |
| Constipation | 3 | 2.1 | – | – |
| Agitation | – | – | 4 | 2.9 |
| Anxiety | – | – | 4 | 2.9 |
| CNS stimulation | – | – | 7 | 5.1[c] |
| Somnolence | 1 | 0.7 | 7 | 5.1 |
| Extra pyramidal syndrome | – | – | 2 | 1.4 |
| Trismus | – | – | 2 | 1.4 |
| Dyskinesia | – | – | 2 | 1.4 |
| Hypertension | 1 | 0.7 | 5 | 3.6 |
| Hypotension | – | – | 3 | 2.2 |

[a]$P = 0.02$; [b]$P = 0.04$; [c]$P = 0.006$.

significant. In 72% of patients receiving granisetron and 80% of patients receiving metoclopramide/dexamethasone combination, no additional anti-emetic therapy was required for the total duration of the study.

### *Clinical and laboratory monitoring*

Mean changes in pulse rate, blood pressure and temperature were small and were not significantly different between groups. At no time did the investigators rate fewer than 79% of patients in the granisetron group and 73% of patients in the comparator group as well. Alertness varied with the patients' sleep patterns and there was no discernable difference between the two groups. Analysis of laboratory investigations again revealed no clinically significant changes in parameters in either group of patients.

### *Adverse events*

During the 7-day period of study one or more adverse events were reported in 33 patients (23%) in the granisetron group and 45 patients (33%) in the metoclopramide/dexamethasone group. This was not statistically different ($P = 0.07$) (Table II). Headache was reported significantly more frequently in the granisetron group with 14 patients (9.8%) experiencing this compared to four patients (2.9%) in the comparator group ($P = 0.02$). This was the commonest adverse event in this treatment group. Headaches were usually mild and resolved either spontaneously or in response to treatment with a mild analgesic such as paracetamol. Other adverse events occurring in three or more patients in this group were diarrhoea and constipation. Significantly more patients in the comparator group reported diarrhoea, 7.2% compared to 2.1% in the granisetron group ($P = 0.04$) and somnolence 5.1% compared to 0.7% ($P = 0.03$) than in the granisetron group. In addition there were significantly more extrapyramidal reactions (including extrapyramidal syndrome, dyskinesia, trismus and CNS stimulation) in the comparator group ($n = 13$) than in the granisetron group where none were reported ($P < 0.05$).

Two patients in the granisetron group experienced serious adverse events during the 7-day course of the study. One patient experienced dyspnoea which resulted in the patient's death but was considered to be unrelated to granisetron therapy. Another reported severe buccal cavity haemorrhage secondary to malignant involvement which was again considered to be unrelated to therapy with granisetron. Four patients in the comparator group experienced serious adverse events. One patient had dyspnoea, tachycardia and pericarditis, a second a transient ischaemic attack, and the third acute respiratory distress and renal insufficiency. None of these events were thought to be related to anti-emetic therapy. A fourth patient experienced a severe extrapyramidal reaction which was considered to be related to treatment with metoclopramide.

### Discussion

The pattern of emesis following chemotherapy is variable and is dependent on several factors, including the chemotherapeutic agent, the patient's previous history of nausea and vomiting, the patient's previous experience of chemotherapy and other patient characteristics (Roila *et al.*, 1985; Levanthal *et al.*, 1988; Andrykowski *et al.*, 1985; D' Aequisto *et al.*, 1986; Morrow, 1982). Nausea and vomiting occurring the first 24 h following chemotherapy (acute emesis) is of particular importance in determining the pattern of emesis. Patients experiencing poor control of acute emesis are more likely to experience delayed emesis (nausea and vomiting after the first 24 h) than patients who are protected during this period (Kris *et al.*, 1985*b*; Roila *et al.*, 1991). Initial control of acute emesis is therefore of importance with drugs known to cause delayed emesis, such as cisplatin. In addition, patients who suffer severe acute or delayed emesis are more likely to suffer anticipatory nausea and vomiting prior to receiving repeat cycles of chemotherapy (Morrow, 1982) which may lead to the patient delaying or refusing further courses of potentially curative treatment (Wilcox *et al.*, 1982). Gaining control of emesis in the first 24-h period following chemotherapy can, therefore, improve the likelihood of successful management of subsequent cycles.

In this single-blind study the objective was to determine the comparative efficacy and safety of granisetron compared with the combination of high-dose metoclopramide and dexamethasone, which is commonly used to treat cisplatin-induced nausea and vomiting. The anti-emetic efficacy of each treatment in the first 24 h following cytostatic therapy was not shown to be significantly different. Seventy percent of patients receiving granisetron and 67% of patients receiving the metoclopramide and dexamethasone combination were complete responders. Thirteen percent of patients in granisetron group and 9% in the comparator group were major responders with major efficacy of each treatment group being 83% and 77% respectively. There were more failures in the comparator gorup (10%) than in the granisetron group (5%) although there was little difference in the number of minor responders in either group. Thus both preparations produced effective control of symptoms during the first 24-h period. Granisetron also appears to have efficacy in resolving or improving symptoms of breakthrough nausea and vomiting. Of 30 patients who required an additional dose of granisetron 87% responded in that their symptoms resolved or improved. It seems, therefore, that granisetron is useful, not only for the prophylaxis of cytostatic-induced emesis, but also for its treatment.

In terms of the time to first symptoms of nausea or vomiting there was also no significant difference between the two groups, and there was no difference between the two treatment groups when assessed for global efficacy by either the patient or the clinician.

A proportion of patients in both treatment groups remained free from further nausea or vomiting in the 6 days following chemotherapy, whereas 36% of granisetron-treated patients and 47% of comparator-treated patients remained complete responders for the whole study period. It is difficult to draw any firm conclusions about this observation which may indicate that both treatments offer some protection against emesis beyond the day of chemotherapy.

The results of this study show that granisetron has a good safety profile with no serious granisetron-related adverse effects. The commonest adverse event was headache which was usually mild and resolved either spontaneously or with mild analgesia. Conversely the use of dopamine antagonists is known to be associated with a number of undesirable side effects as was seen in this study where 13 extrapyramidal reactions were reported, one of which was serious. This particular problem has been reported as occurring in up to 30% of patients taking dopamine antagonists (Kris *et al.*, 1983). The effect is seen most frequently in younger patients and with increasing doses, precluding the use of these drugs at high doses for long periods of time (Kris *et al.*, 1983). This effect can be very distressing to the patient and may itself require treatment, adding further complication to an already cumbersome administration schedule. Granisetron does not cause extrapyramidal symptoms. This is related to the fact that it is a highly selective 5-$HT_3$ receptor antagonist and that it has no interaction with the dopamine receptor. Indeed, it has little or no interaction with many other receptor sites (Bermudez *et al.*, 1988).

In addition, somnolence which was seen in 5.1% of comparator-treated patients was only observed in one patient (0.7%) treated with granisetron. Drowsiness is an undesirable side effect as patients may be unable to drive home after therapy and have to be escorted or remain in the clinic. Diarrhoea also occurred significantly more frequently in the comparator group compared with the granisetron group.

In conclusion, granisetron, when given as a convenient single 5-min infusion (40 $\mu g\,kg^{-1}$, i.v.) provided antiemetic protection comparable to that given by standard anti-emetic regimens, with the significant advantage of simplicity of administration. Granisetron is also safe to administer and is not associated with extrapyramidal effects which were associated with the comparator regimen. In addition, patients experienced significantly less side effects such as diarrhoea and somnolence with granisetron compared with the comparator.

### References

ASSOCIATION OF THE BRITISH PHARMACEUTICAL INDUSTRY (1989). ABPI data sheet compendium 1989–1990. DataPharm Publications.

ANDRYKOWSKI, M.D., REDD, W.H. & HATFIELD, A.L. (1985). Development of anticipatory nausea – a prospective analysis. *J. Consult. Clin. Psychol.*, **3**, 447–454.

BARDFIELD, P.A. (1966). A controlled double-blind study of trimethobenzamide, prochlorperazine and placebo. *JAMA*, **196**, 796–798.

BERMUDEZ, J., BOYLE, E.A., MINER, M.D. & SANGER, G.J. (1988). The antiemetic potential of the 5-hydroxytryptamine receptor antagonist BRL.43694. *Br. J. Cancer*, **58**, 644–650.

COATES, A., ABRAHAM, S., KAYE, S.B., SOWERBUTTS, T., FREWIN, C., FOX, R.M. & TATTERSALL, M.H. (1983). On the receiving end – patient perception of the side-effects of cancer chemotherapy. *Eur. J. Cancer Clin. Oncol.*, **19** (suppl 2), 203–208.

D'ACQUISTO, R.W., TYSON, L.B., GRALLA, R.J., CLARK, R.A., KRIS, M.G., VON WITTE, D.M. & CACAVIO, A. (1986). The influence of a chronic high alcohol intake on chemotherapy induced nausea and vomiting. *Proc. Am. Soc. Clin. Oncol.*, **5**, 257.

GRALLA, R.J., ITRI, L.M., PISKO, S.E., SQUILLANTE, A.E., KELSEN, D.P., BRAUN, D.W.Jr, BORDIN, L.A., BRAUN, T.J. & YOUNG, C.W. (1981). Antiemetic efficacy of high dose metoclopramide – randomised trials with placebo and prochlorperazine in patients with chemotherapy-induced nausea and vomiting. *N. Engl. J. Med.*, **305**, 905–909.

GRUNBERG, S.M., AKERLEY, W.L., KRAILO, M.D., JOHNSON, K.B., BAKER, C.R. & CARIFFE, P.A. (1986). Comparison of metoclopramide and metoclopramide plus dexamethasone for complete protection from cisplatinum induced emesis. *Cancer Invest.*, **4**, 379–385.

KRIS, M.G., TYSON, L.B. & GRALLA, R.J. (1983). Extrapyramidal reactions with high dose metoclopramide. *N. Engl. J. Med.*, **309**, 443–444.

KRIS, M.G., GRALLA, R.J., CLARK, R.A., TYSON, L.B., FIORE, J.J. & KELSEN, D.P. (1985*a*). Consecutive dose finding trials adding lorazepam to the combination of metoclopramide plus dexamethasone. Improved subjective effectiveness over the combination of diphenhydramine plus metoclopramide plus dexamethasone. *Cancer Treat. Rep.*, **69**, 1257–1262.

KRIS, M.G., GRALLA, R.J., CLARK, R.A., TYSON, L.B. & GROSHEN, S. (1987). Antiemetic control and prevention of side-effects of anticancer therapy with lorazepam and diphenhydramine when used in combination with metoclopramide and dexamethasone: a double-blind randomised trial. *Cancer*, **60**, 2816–2822.

KRIS, M.G., GRALLA, R.J., CLARK, R.A., TYSON, L.B., O'CONNELL, J.P., WERTHEIM, M.S. & KELSEN, D.P. (1985*b*). Incidence course and severity of delayed nausea and vomiting following the administration of high dose cisplatin. *J. Clin. Oncol.*, **3**, 1379–1384.

LASZLO, J. (1983). Emesis as limiting toxicity in cancer chemotherapy. In *Anti-emetics and Cancer Chemotherapy*, Laszlo, J. (ed.) pp. 1–5. Williams & Williams, Philadelphia.

LEVENTHAL, H., EASTERLING, D.V., NERENZ, D.R. & LOVE, R.R. (1988). The role of motion sickness in predicting anticipatory nausea. *J. Behav. Med.*, **11** (suppl 2), 117–130.

MARTY, M. ON BEHALF OF THE GRANISETRON STUDY GROUP (1990). A comparative study of the use of granisetron, a selective $5HT_3$ antagonist, versus a standard antiemetic regimen of chlorpromazine plus dexamethasone in the treatment of cytostatic-induced emesis. *Eur. J. Cancer*, **26** (suppl 1), S29–S33.

MINER, W.D. & SANGER, G.J. (1986). Inhibition of cisplatin induced vomiting by selective 5-hydroxytryptamine M receptor antagonism. *Br. J. Pharmacol.*, **88**, 497–499.

MOERTEL, C.G. & REITEMEIER, R.J. (1969). Controlled clinical studies of orally administered anti-emetic drugs. *Gastroenterology*, **57**, 262–268.

MORROW, G.R. (1982). Prevalence and correlates of anticipatory nausea and vomiting in chemotherapy patients. *J. Natl Cancer Inst.*, **68**, 585–588.

ROILA, F., TONATO, M., BASURTO, C., CANALETTI, R., MORSIA, D., PASSALACQUA, R., DICOSTANZO, F., DONATI, D., COLOMBO, N. & BALLATORI, E. (1985). Antiemetic activity on two different high doses of metoclopramide in cisplatin treated patients. *Cancer Treat. Rep.*, **69**, 1353–1357.

ROILA, F., BOSCHETTI, E., TONATO, M., BASURTO, C., BRACARDA, S., PICCIAFUOCO, M., PATOIA, L., SANTI, E., PENZA, O., BALLATORI, E. & DEL FAVERO, A. (1991). Predictive factors of delayed emesis in cisplatin treated patients and antiemetic activity and tolerability of metoclopramide or dexamethasone. *Am. J. Clin. Oncol.*, **14**, 238–242.

SMITH, I.E. ON BEHALF OF THE GRANISETRON STUDY GROUP. (1990). The comparison of two dose levels of granisetron in patients receiving moderately emetogenic cytostatic chemotherapy. *Eur. J. Cancer*, **26** (suppl 1), S19–S22.

SOUKOP, M. ON BEHALF OF THE GRANISETRON STUDY GROUP. (1990). A dose-finding study of granisetron in patients receiving high dose cisplatin. *Eur. J. Cancer*, **26** (suppl 1), S16–S20.

WILCOX, P.M., FETTING, J.H., NETTESHEIM, K.M. & ABELOFF, M.D. (1982). Anticipatory vomiting in women receiving cyclophosphamide, methotrexate, and 5-FU (CMF) adjuvant therapy for breast carcinoma. *Cancer Treat. Rep.*, **66**, 1601–1604.